# Learning and Development among Care Workers for Older Adults in Finland

**DOI:** 10.1093/geroni/igaf122.4150

**Published:** 2025-12-31

**Authors:** Younho Lee, Yongho Chon

**Affiliations:** Incheon National University, Incheon, Republic of Korea; Incheon National University, Incheon, Republic of Korea

## Abstract

Expanding learning perspectives can give insight into the mechanisms of developing care workers for older adults and organizations. Care workers learn from lecture-based education and training regarding health and social care while learning from hands-on experiences by observing, asking questions, and interacting with skilled colleagues (Dewing, 2010; Raymond et al., 2014). That is, they experience various types of learning because of the influence of context, relationship, and interaction at the workplace (Marsick & Watkins, 1990; Eraut, 2004; Anvik et al., 2020). However, most interventions in learning are education and training, and these methods are difficult to assess direct effectiveness (Surr et al., 2017, 2019; Latham et al., 2024). Therefore, this study aims to examine the experiences and perspectives of care workers’ learning in Siun Sote, Finland. A qualitative study was conducted at the Siun sote center. Eight nurses, seven care assistants, two social workers, three registered nurses, and three managers participated in the interviews. The main finding is as follows. First, care workers participated in organization-led training and obtained knowledge and skills in health and social care. Second, care workers experienced observation, supervision, and delegation, and achieved person-centered care, problem-solving, and situational judgment competencies. Care workers participated in teamwork and multidisciplinary collaboration. Siun sote runs knowledge management systems, organizational training programs, and task guidelines. The organization can strengthen its capacities to provide diverse services for its clients and manage knowledge and skills. This study shed light on various types of learning activities and outcomes for care workers and the organization itself.

